# Integrative transcriptome and proteome analyses of clear cell renal cell carcinoma develop a prognostic classifier associated with thrombus

**DOI:** 10.1038/s41598-023-36978-5

**Published:** 2023-06-16

**Authors:** Xiaolei Shi, Qingyang Pang, Xinwen Nian, Aimin Jiang, Haoqing Shi, Wenqiang Liu, Xinxin Gan, Yisha Gao, Yiren Yang, Jin Ji, Xiaojie Tan, Chengwu Xiao, Wei Zhang

**Affiliations:** 1 Department of Urology, Changhai Hospital, Naval Medical University, 168 Changhai Rd, Shanghai, 200433 China; 2 Department of Pathology, Changhai Hospital, Naval Medical University, 168 Changhai Rd, Shanghai, 200433 China; 3 Department of Epidemiology, Naval Medical University, 800 Xiangyin Rd, Shanghai, 200433 China

**Keywords:** Renal cell carcinoma, Cancer genomics

## Abstract

Clear cell renal cell carcinoma (ccRCC) with venous tumor thrombus (VTT) is associated with poor prognosis. Our integrative analyses of transcriptome and proteome reveal distinctive molecular features of ccRCC with VTT, and yield the development of a prognostic classifier to facilitate ccRCC molecular subtyping and treatment. The RNA sequencing and mass spectrometry were performed in normal-tumor-thrombus tissue triples of five ccRCC patients. Statistical analysis, GO and KEGG enrichment analysis, and protein–protein interaction network construction were used to interpret the transcriptomic and proteomic data. A six-gene-based classifier was developed to predict patients’ survival using Cox regression, which was validated in an independent cohort. Transcriptomic analysis identified 1131 tumorigenesis-associated differentially expressed genes (DEGs) and 856 invasion-associated DEGs. Overexpression of transcription factor EGR2 in VTT indicated its important role in tumor invasion. Furthermore, proteomic analysis showed 597 tumorigenesis-associated differentially expressed proteins (DEPs) and 452 invasion-associated DEPs. The invasion-associated DEPs showed unique enrichment in DNA replication, lysine degradation, and PPAR signaling pathway. Integration of transcriptome and proteome reveals 142 tumorigenesis-associated proteins and 84 invasion-associated proteins displaying changes consistent with corresponding genes in transcriptomic profiling. Based on their different expression patterns among normal-tumor-thrombus triples, RAB25 and GGT5 were supposed to play a consistent role in both tumorigenesis and invasion processes, while SHMT2 and CADM4 might play the opposite roles in tumorigenesis and thrombus invasion. A prognostic classifier consisting of six DEGs (DEPTOR, DPEP1, NAT8, PLOD2, SLC7A5, SUSD2) performed satisfactorily in predicting survival of ccRCC patients (HR = 4.41, *P* < 0.001), which was further validated in an independent cohort of 40 cases (HR = 5.52, *P* = 0.026). Our study revealed the transcriptomic and proteomic profiles of ccRCC patients with VTT, and identified the distinctive molecular features associated with VTT. The six-gene-based prognostic classifier developed by integrative analyses may facilitate ccRCC molecular subtyping and treatment.

## Introduction

Metastatic clear cell renal cell carcinoma (ccRCC) is the most lethal urological cancer worldwide. The primary tumor invades the venous system and forms a renal vein or inferior vena cava thrombus in approximately 4–15% of RCC patients^1,2^. As venous tumor thrombus (VTT) is a potentially key initial stage of metastatic ccRCC, patients with VTT exhibit poor prognosis if left untreated, with a median survival of 5 months, and a 1-year disease-specific survival rate of 29%^3,4^. Although advances in surgical management have improved the survival rate among ccRCC patients with VTT^5^, the challenges of postoperative complications and perioperative mortality remain, and limited preoperative clinical benefit is derived from targeted therapy^6^. Early detection of VTT formation and development, and understanding underlying genomic features are thus essential.

Previous studies have suggested that ccRCC exhibits extensive functional and genomic intratumoral heterogeneity^7,8^ shaped by spatial niches^9,10^. The results of multiregion whole exome sequencing indicate that ccRCC-associated VTT contain viable tumor cells with mutational heterogeneity compared to the primary tumor^11^. However, the molecular features and protein expression profiles in VTT remain unclear. Thus, there is an urgent need to understand the unique biological features of VTT through integrated transcriptomic and proteomic analyses.

Proteomics offers many advantages, including wide proteome coverage and ideal analytical robustness^12,13^, and is increasingly being applied to studies investigating biological mechanisms and developing clinical applications. To date, few published studies have investigated for correlation between deregulated transcriptomic and proteomic homeostasis in ccRCC and VTT formation. We herein report transcriptomic and proteomic analyses of thrombus, tumor, and normal tissues from patients with ccRCC to reveal distinctive molecular features of VTT. Further bioinformatics and survival analyses identified novel targets for molecular classification, prognosis prediction, and drug development.

## Methods

### Clinical sample collection and storage

**Figure 1:**
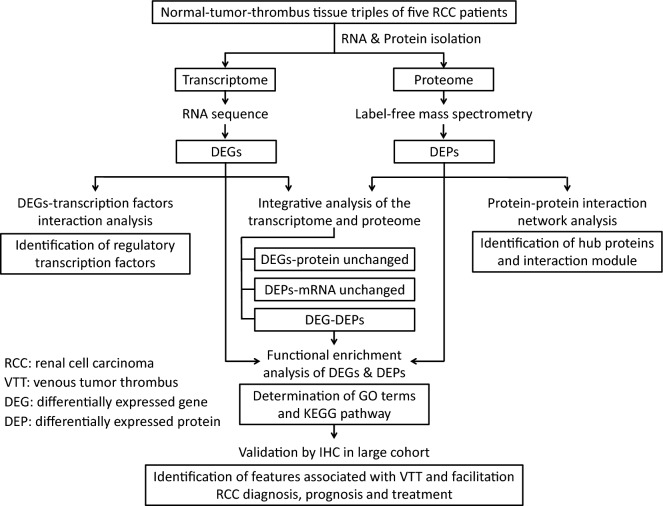
Flowchart of transcriptomic and proteomic analyses in ccRCC patients with VTT.

The workflow of transcriptomic and proteomic studies to reveal features of ccRCC associated with VTT is summarized in Fig. 1. This study was approved by the Ethics Review Committee of Changhai Hospital, Naval Medical University. Written informed consent was obtained from each patient before any study procedures were performed. All methods in our study were performed in accordance with relevant guidelines and regulations. Patients were included if they had histologically confirmed ccRCC with VTT and had received no treatment prior to surgery. The thrombus, tumor, and normal tissues were obtained from each patient after nephrectomy and tumor thrombus resection. Specimens were collected immediately after operation, and snap-frozen in liquid nitrogen until further processing. Transcriptomic and proteomic services were provided by Shanghai Luming Biological Technology Co., LTD (Shanghai, China).

### Analyses of differentially expressed genes/proteins (DEGs/DEPs)

DEG and DEP analyses were performed using the “limma” package in R statistical software. DEGs and DEPs were identified for three comparisons: RCC versus normal renal tissue (NRT), VTT versus NRT, and VTT versus RCC. DEGs/DEPs were defined as |log2 FC|> 2 and *P* < 0.05. Gene Ontology (GO) functional annotation and Kyoto Encyclopedia of Genes and Genomes (KEGG) pathway enrichment were performed in R using the “clusterProfiler” package. Bar plots, dot plots, and chord plots were constructed to visualize functional annotation results via the “GOplot” package in R.

### Screening for genes associated with survival prognosis

Univariate Cox regression analysis was performed to identify key genes associated with overall survival (OS) using the survival package (version 3.4–0; https://CRAN.R-project.org/package=survival). The e1071 package (version 1.7–13; https://CRAN.R-project.org/package=e1071) and caret package (version 6.0–93; https://CRAN.R-project.org/package=caret) were used to further screen for an optimal prognostic gene set for OS using the SVM-RFE method, which is an iterative backward selection algorithm that can recursively remove one feature gene with the smallest ranking score until the optimal feature gene set remains. Subsequently, an SVM classifier was constructed to predict OS based on expression levels of genes in the optimal prognostic gene set. The external TCGA-KIRC dataset was used to verify the results of SVM classification analysis.

### Development and validation of a prognostic classifier for survival

Multivariate Cox regression analysis was performed to identify independent prognostic genes for OS using the Survival Package (version 3.3.1; https://CRAN.R-project.org/package=survival). A risk score model of prognostic mRNA markers was established using the following formula: risk score = ∑βDEGs × ExpDEGs. DEGs represent the estimated contribution coefficient of independent prognostic mRNAs in multivariate Cox regression analysis, and ExpDEGs denote the level of independent prognostic genes. All patients were then divided into high- or low-risk groups with the median risk score as the cutoff value. The difference in survival between the two groups was shown using Kaplan–Meier curves. To evaluate the predictive value of this model, time-dependent ROC curves for 1-year, 3-year, and 5-year survivals were drawn to obtain area under the curve (AUC) values.

### Statistical analyses

An adjusted *P* < 0.05 was considered statistically significant, and all data in this research is displayed as means ± standard deviation. Experimental results were calculated and visualized using R 4.1.2 and GraphPad Prism 6.

### Detailed experimental materials and methods

Detailed descriptions of the experimental procedures and statistical analyses are provided in the Supplemental Materials and Methods.

### Ethics approval and consent to participate

The study protocol was reviewed and approved by the Ethics Review Committee of Changhai Hospital. Written informed consent was obtained from each patient before any study procedures were performed.

## Results

### Patient and sample characteristics

Five patients with ccRCC associated VTT were included in this study. Their baseline characteristics are presented in Table S1. Respective mean renal mass diameter and thrombus length values were 8.8 ± 3.6 cm and 6.0 ± 3.1 cm. Among the included patients, one had a renal vein tumor thrombus with a length of 2 cm (the shortest), and the remaining four had inferior vena cava tumor thrombi with lengths of 5–10 cm (Figure S1A). RNA sequencing and mass spectrometry revealed a high correlation between RNA (r = 0.94) and protein (r = 0.86) expression in renal tumor and thrombus tissues (Figure S1).

### Transcriptomic profiling of ccRCC with VTT

**Figure 2:**
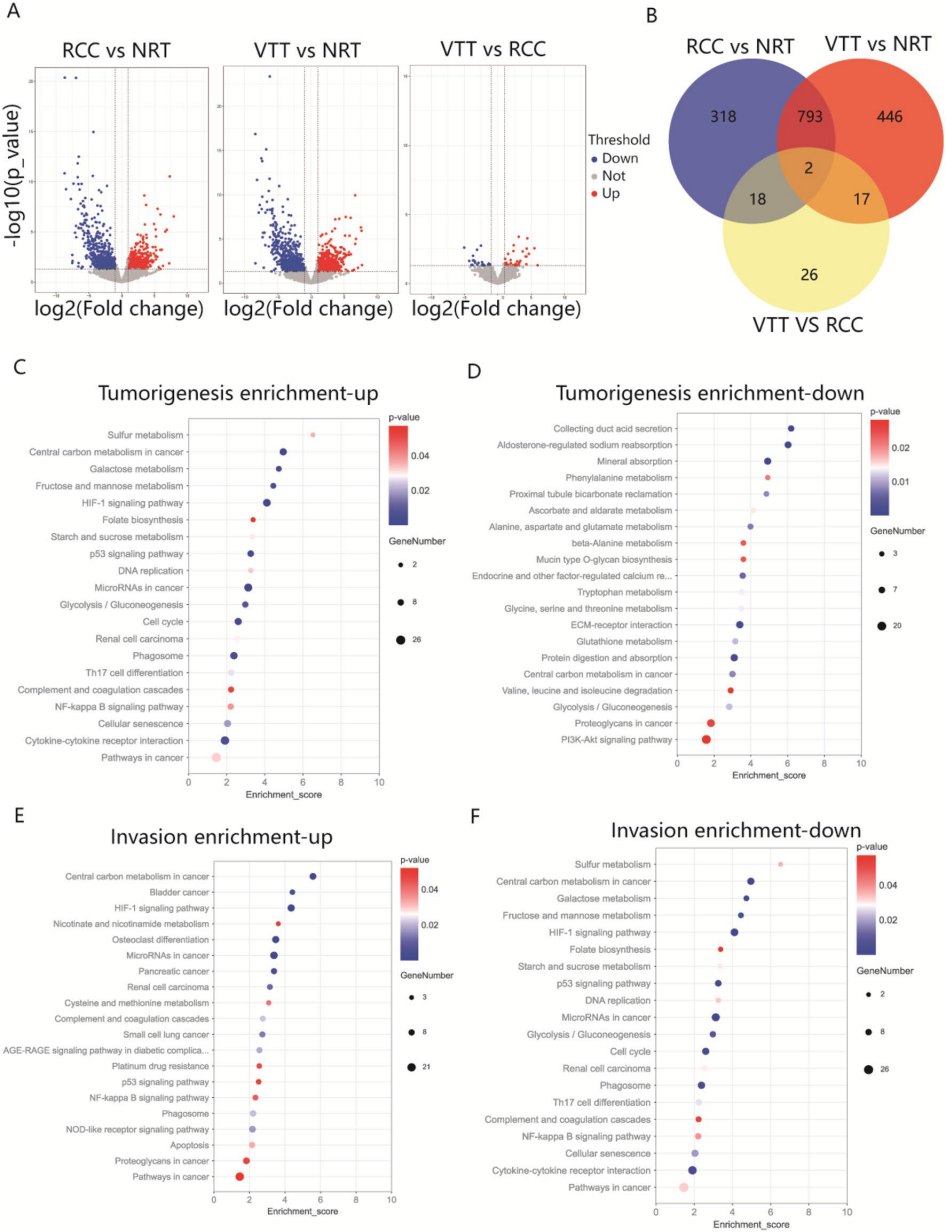
The transcriptomic data of ccRCC associated with VTT. (**A**) Volcano plots of all DEGs in transcriptome analysis of normal, tumor and thrombus tissue. (**B**) Venn diagram to illustrate the overlap of DEGs in different comparisons. (**C**, **D**) KEGG pathway annotation of the tumorigenesis-associated genes. (**E**, **F**) KEGG pathway annotation of the invasion-associated genes.

A pooled analysis of RNA-seq results for matched RCC, VTT, and NRT revealed 1131, 1258, and 63 transcripts that were differentially expressed in RCC versus NRT, VTT versus NRT, and VTT versus RCC groups, respectively (Fig. 2A). The 1131 DEGs in RCC versus NRT were defined as tumorigenesis-associated genes. Among them were 505 up-regulated and 626 downregulated genes. 795 DEGs identified in both RCC versus NRT and VTT versus NRT, and 63 DEGs identified in VTT versus RCC were defined as thrombus invasion-associated genes. The final number of genes associated with thrombus invasion was 856 owing to two overlapping genes. Among these, 382 are up-regulated, and 474 are downregulated (Fig. 2B). Among the tumorigenesis-associated genes, KEGG analysis revealed that the upregulated genes, including SLC7A5, E2F1, HIF1A, VEGFA, and EGFR, were predominantly related to central carbon metabolism in cancer, the cell cycle, and the HIF-1 signaling pathway (Fig. 2C). Downregulated genes including NAT8 and GGT5 were predominantly associated with glutathione metabolism (Fig. 2D). Invasion-associated genes were found to share more than half of their pathways with the tumorigenesis-associated genes, including central carbon metabolism in cancer, and the HIF-1 signaling pathway. We also found some pathways that were specific to invasion-associated genes, such as NLRP3, which is enriched in the NOD-like receptor signaling pathway (Fig. 2E, F).

GO enrichment analysis was also performed to determine the functions of highly significant DEGs. The upregulated genes were significantly enriched for GO terms associated with positive regulation of mast cell chemotaxis (GO:0,060,754), whereas the downregulated genes were significantly enriched for GO terms associated with multicellular organismal water homeostasis (GO:0,050,891). Interestingly, among both tumorigenesis- and invasion-associated DEG sets, the upregulated genes had more enriched molecular functions than the downregulated genes (Figure S3). Transcription factor analysis was conducted on genes involved in tumorigenesis and thrombus invasion using the hTF-target database (http://bioinfo.life.hust.edu.cn/hTFtarget#!/). We screened DEGs for potential transcription factors and identified six transcription factors with significant differences and important functions. These results suggest that MAZ, HIF1A, and E2F1 play roles in both tumorigenesis and invasion, ETV7 plays a role in tumorigenesis, and EGR2 and KLF4 play roles in thrombus invasion (Figure S4).

### Proteomic profiling of ccRCC with VTT

**Figure 3:**
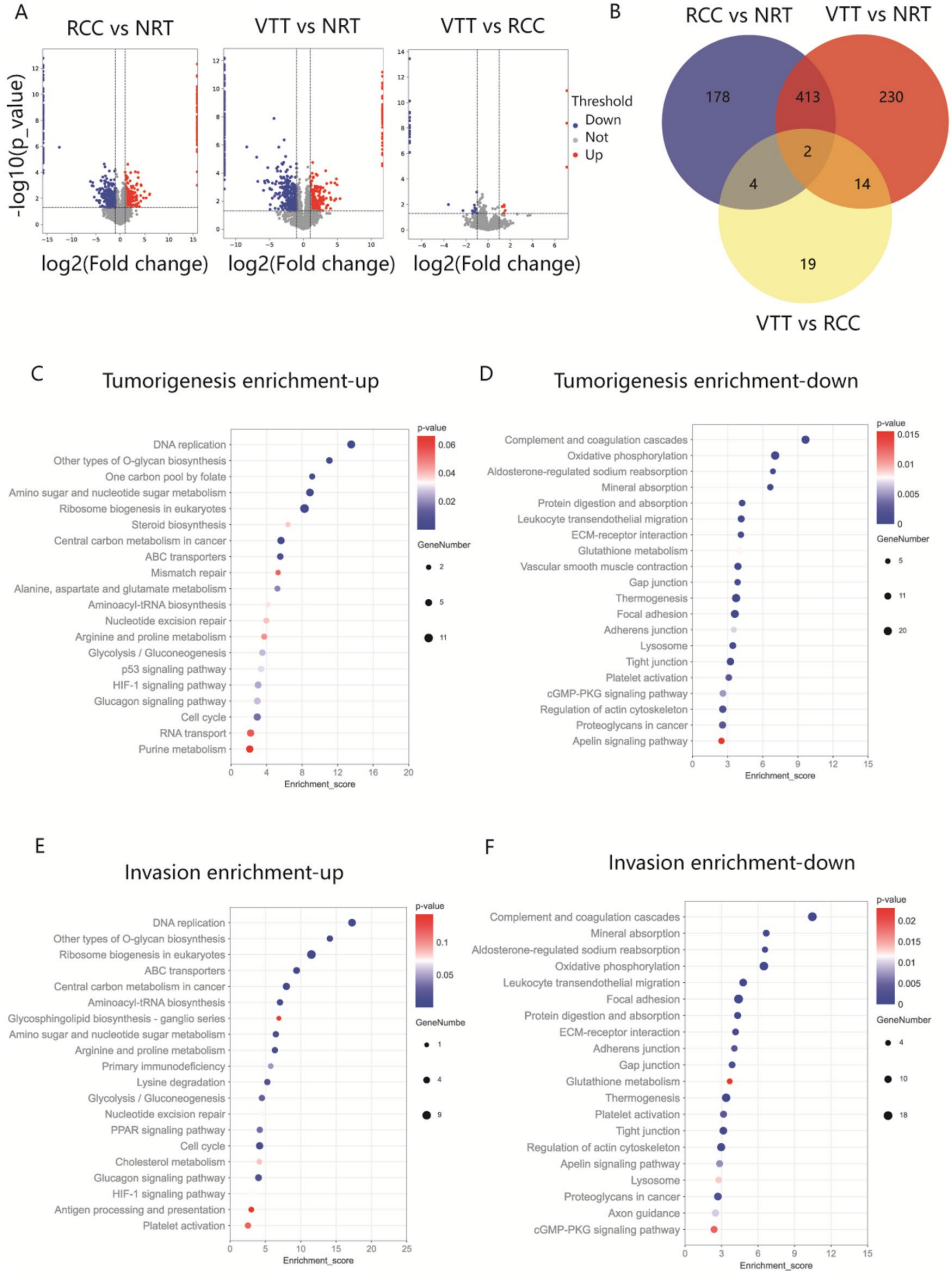
The proteomic data of ccRCC associated with VTT. (**A**) Volcano plots of all DEPs in proteome analysis of normal, tumor and thrombus tissue. (**B**) Venn diagram to illustrate the overlap of DEPs in different comparisons. (**C**, **D**) KEGG pathway annotation of the tumorigenesis-associated proteins. (**E**, **F**) KEGG pathway annotation of the invasion-associated proteins.

Proteomic analysis of matched RCC, VTT, and NRT samples revealed 597, 659, and 39 DEPs in RCC versus NRT, VTT versus NRT, and VTT versus RCC comparisons, respectively (Fig. 3A). A total of 597 DEPs in RCC versus NRT were defined as tumorigenesis-associated proteins, among which 272 were up-regulated and 325 were downregulated. 415 DEPs identified in both RCC versus NRT and VTT vs. NRT comparisons, and 39 DEPs identified in VTT versus RCC were defined as thrombus invasion-associated proteins. The final number of proteins associated with thrombus invasion was 452 owing to two overlapping proteins. Among these, 157 were up-regulated, and 295 were downregulated (Fig. 3B). Among the tumorigenesis-associated proteins, KEGG analysis revealed that the upregulated proteins, including SLC7A5 and PYCR1, were predominantly related to central carbon metabolism and arginine and proline metabolism (Fig. 3C), and the downregulated proteins were related to focal adhesion and tight junctions (Fig. 3D). Among invasion-associated proteins, upregulated proteins, including PLOD2 and FABP5, were predominantly associated with lysine degradation, the PPAR signaling pathway, and DNA replication (Fig. 3E), whereas downregulated proteins, including GGT5, were predominantly associated with glutathione metabolism (Fig. 3F).

GO enrichment analysis revealed overlap in GO terms between tumorigenesis- and invasion-associated proteins. The upregulated genes were significantly enriched for GO terms associated with the nucleolus (GO:0,005,730) and membrane (GO:0,016,020), whereas the downregulated genes were significantly enriched for GO terms associated with extracellular exosomes (GO:0,070,062) and cell adhesion (GO:0,007,155). Invasion-associated proteins showed unique enrichment in GO terms including positive regulation of protein binding (Figure S5). After GO and KEGG enrichment analyses, DEPs with significant functions and non-zero expression were used to construct a PPI network of tumorigenesis- and invasion-associated proteins (PI score ≥ 0.9). SLC1A5, SLC7A5, PYCR1, RAB25, DPEP1, and GGT5 were the central hubs of this network (Figure S5). With a high degree of connectivity between the processes of tumorigenesis and thrombus invasion, SLC1A5, SLC7A5, and PYCR1 were identified as hub upregulated proteins, and DPEP1 and GGT5 were hub downregulated proteins.

### Effects of DEGs on tumorigenesis and thrombus invasion

**Figure 4:**
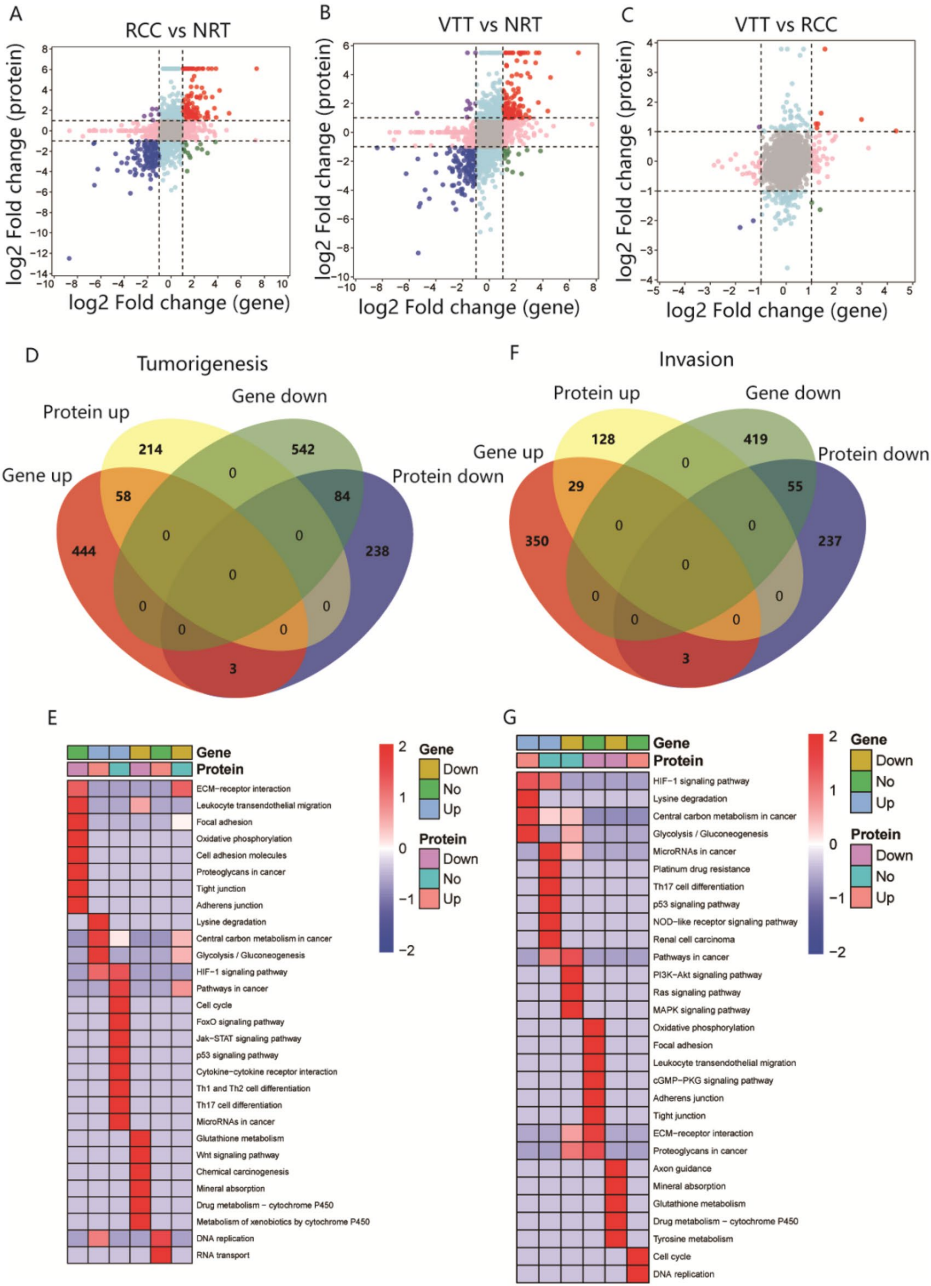
The integrative transcriptomic and proteomic data of ccRCC associated with VTT. (**A**, **B** and **C**) Scatter plots of the correlations between expressions of DEPs and DEGs in RCC versus NRT, VTT versus NRT, and VTT versus RCC. (**D**) Venn diagram to illustrate the consistently and differentially expressed genes/proteins and (**E**) associated KEGG pathway annotation in tumorigenesis. (**F**) Venn diagram to illustrate the consistently and differentially expressed genes/proteins and (**G**) associated KEGG pathway annotation in thrombus invasion.

To identify key genes and protein pathways involved in tumorigenesis and thrombus invasion, we integrated gene-level mRNA expression data with quantitative proteomics. Only a small fraction of genes displayed disagreement between the changes in their mRNA and protein levels, with 93.1% (284/305) consistently identified in RCC versus NRT (Fig. 4A), 91.9% (294/320) consistently identified in VTT versus NRT (Fig. 4B), and 75% (9/12) consistently identified in VTT versus RCC (Fig. 4C). These results suggest good consistency between transcriptome and proteome. Two gene sets, tumorigenesis and thrombus invasion, were screened according to previous criteria. In the tumorigenesis-associated gene set, 142 proteins displayed the same trend in abundance as their corresponding genes in transcriptomic profiling (Fig. 4D). Kyoto Encyclopedia of Genes and Genomes (KEGG) analysis divided all significantly altered genes into six clusters based on RNA/protein expression in both omics to determine their biological functions. The upregulated genes were enriched in central carbon metabolism in cancer, Glycolysis/Gluconeogenesis, DNA replication, the HIF-1 signaling pathway, and lysine degradation, and the downregulated genes were enriched in leukocyte transendothelial migration and Wnt signaling (Fig. 4E). In the invasion assocated gene set, 84 proteins showed a similar trend in abundance at transcriptome and proteome levels (Fig. 4F). The KEGG pathways associated with the upregulated genes were similar to those associated with the tumorigenesis-associated gene set, including Glycolysis/Gluconeogenesis, Central carbon metabolism in cancer, and the HIF-1 signaling pathway, and the downregulated genes were enriched in glutathione metabolism and axon guidance (Fig. 4G).

**Figure 5:**
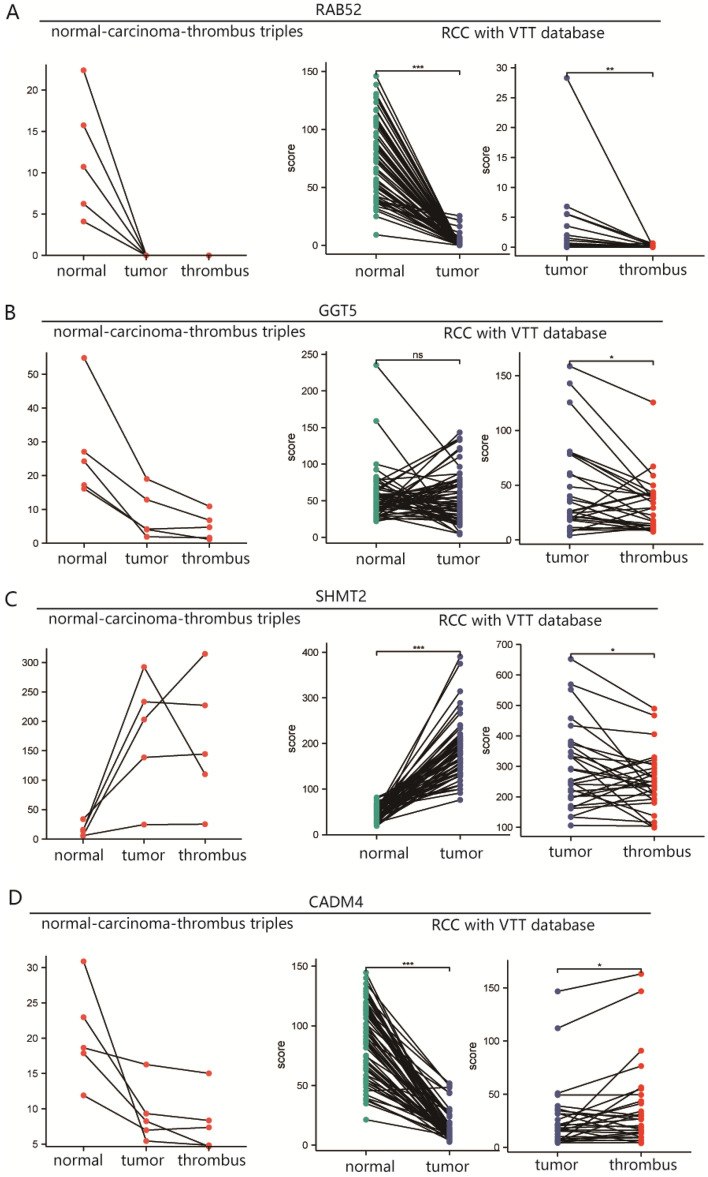
Expression change trends of the dysregulated genes such as (**A**) RAB25, (**B**) GGT5, (**C**) SHMT2, and (**D**) CADM4 among normal, tumor and thrombus tissue based on our own and Wang’s external datasets. Each line represented a case. *p < 0.05, **p < 0.01, ***p < 0.001.

After their identification by KEGG and GO analyses, the expression of hub genes, including RAB25, GGT5, SHMT2, and CADM4, was verified using the dataset obtained by Wang et al.^14^ for external validation. RAB25 and GGT5 expression progressively decreased from normal tissue to tumor to thrombus, suggesting they consistently exert suppressive roles in both tumorigenesis and invasion (Fig. 5A, B). Interestingly, SHMT2 expression was upregulated in the RCC group and further downregulated in the VTT group, whereas CADM4 expression changed in the opposite direction, indicating that SHMT2 and CADM4 may play opposing roles in tumorigenesis and thrombus invasion (Fig. 5C, D).

### Survival prediction using a prognostic classifier associated with thrombi

**Figure 6:**
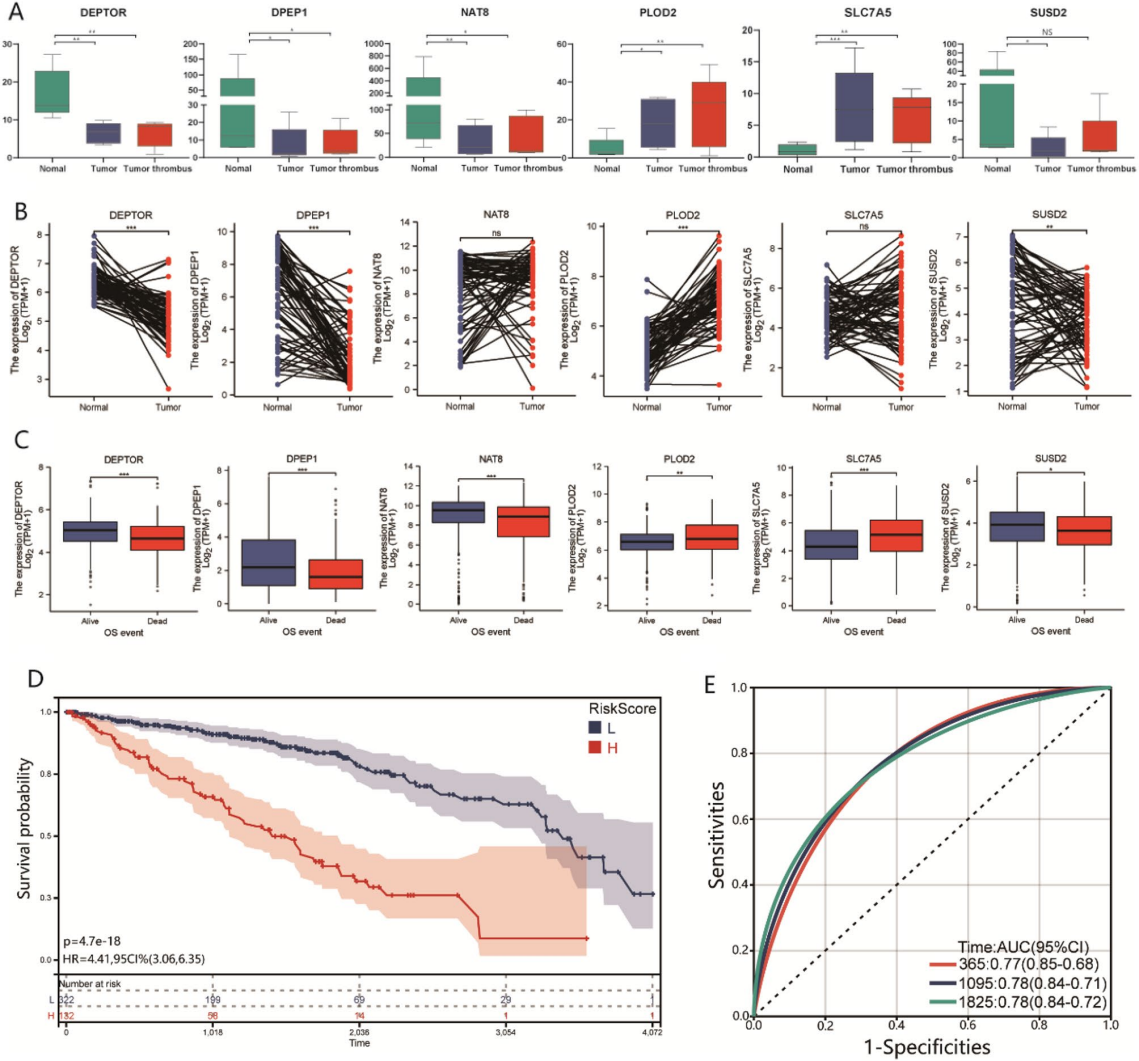
The establishment of a prognostic classifier associated with thrombus. (**A** and **B**) Expression change trends of the six hub genes among normal, tumor and thrombus tissue based on our own dataset and Wang’s external dataset. (**C**) The different expressions of the six hub genes between ccRCC patients with different OS events based on TCGA database. (**D**) The Kaplan–Meier curves of OS for ccRCC patients in high-risk and low-risk groups by prognostic classifier in TCGA database. (**E**) The receiver operating characteristic curves for the prognostic classifier predicting OS of ccRCC patients. *p < 0.05, **p < 0.01, ***p < 0.001.

Six hub genes associated with survival were used to establish a prognostic classifier (Figure S6). Among these six hub genes, DEPTOR, DPEP1, NAT8, and SUSD2 were expressed at their highest levels in normal tissues, whereas PLOD2 and SLC7A5 were expressed at their highest levels in tumor thrombi (Fig. 6A). The expression of these genes was externally validated using Wang’s dataset^14^, confirming that the expression differences in DEPTOR, DPEP1, PLOD2, and SUSD2 between normal and tumor tissues were consistent with our data (Fig. 6B). According to the (TCGA) database, significant expression differences in these hub genes could also be seen between patients with different OS events (366 alive vs. 173 dead). In patients with shorter survival times, DEPTOR, DPEP1, NAT8, and SUSD2 were overexpressed, and PLOD2 and SLC7A5 expression was suppressed (All *P* < 0.05; Fig. 6C). We calculated the risk score for survival in each case according to the expression levels of these six genes, then divided the patients into high- or low-risk groups. We found that patients in the high-risk group had shorter survival times (*P* < 0.001; Fig. 6D). Furthermore, a panel of six survival-related DEGs performed satisfactorily as a prognostic classifier in predicting the prognosis of patients with ccRCC (1-year AUC = 0.77; 3-year AUC = 0.78; 5-year AUC = 0.78) (Fig. 6E).

**Figure 7:**
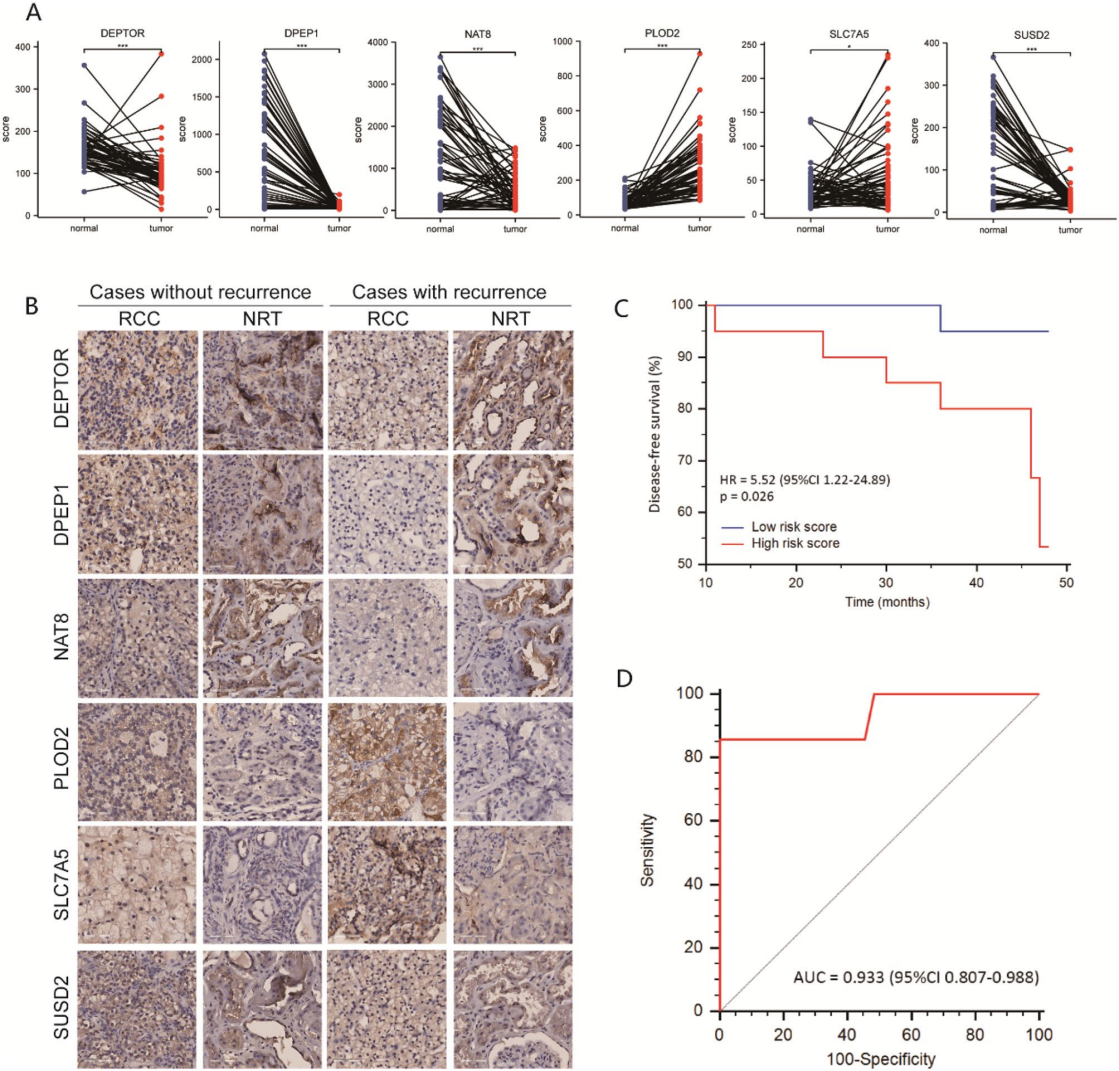
The validation for clinical application of the prognostic classifier associated with thrombus. (**A**) Differences in protein expressions of the six hub genes between normal and tumor tissues based on Wang’s external dataset. (**B**) Immunohistochemistry analysis of the six hub proteins in tumor tissue of ccRCC patients. (**C**) The Kaplan–Meier curve for disease-free survival of ccRCC patients with IHC-based high- or low-risk scores in a cohort of 40 cases. (**D**) The receiver operating characteristic curve for the IHC-based prognostic classifier predicting 3-year disease-free survival of ccRCC patients. *p < 0.05, **p < 0.01, ***p < 0.001.

Differences in protein expression among these survival-related DEGs between normal and tumor tissues were externally confirmed using Wang’s dataset^14^ (Fig. 7A). The clinical efficacy of the prognostic classifier was validated by immunohistochemistry (IHC) analysis of a tissue microarray from a cohort of 40 patients (Fig. 7B). During a median follow-up of 45 (IQR 45–47) months, seven (17.5%) patients experienced recurrence or metastasis. A Kaplan–Meier curve showed that patients with IHC-based high-risk scores had shorter disease-free survival than those with low-risk scores (HR = 0.181, *P* = 0.026) (Fig. 7C). In addition, the AUC of the IHC-based prognostic classifier for 3-year disease-free survival was 0.933 (95% CI 0.807–0.988) (Fig. 7D).

## Discussion

Although successful advances in targeted drugs and immune checkpoint inhibitors have been achieved for various solid tumors including ccRCC^15^, the efficacy of neoadjuvant and adjuvant therapies in ccRCC patients with VTT remains uncertain. Previous studies have indicated that VTT might act as a metastatic reservoir in some patients, consistent with the poor outcomes associated with untreated thrombi^16,17^. VTT associated with metastases is characterized by a higher grade, mTOR activation, and a particular immune context, but the determining factors for tumor infiltration and metastasis capability have not been thoroughly elucidated^18^. Yue et al. revealed that macrophages, malignant cells, endothelial cells, and myofibroblasts exhibit enhanced extracellular matrix remodeling capacity in tumor thrombi, which is associated with poor survival^19^. However, the detailed metastasis-promoting mechanisms and molecular features of thrombi are not yet fully understood^14^. In this study, we investigated the transcriptomic and proteomic profiles of ccRCC tumors and thrombi to provide an overview of their distinctive molecular features. Integration of transcriptome and proteome data revealed that RAB25 and GGT5 play consistent roles in both tumorigenesis and invasion, while SHMT2 and CADM4 might exert opposite effects in these two processes. A six-gene-based prognostic classifier consisting of DEPTOR, DPEP1, NAT8, PLOD2, SLC7A5, and SUSD2 was also created, with satisfactory performance in predicting the survival of patients with ccRCC. Our findings not only provide vital information for mechanistic research, but also reveal novel biomarkers for molecular subtyping or therapeutic decision-making.

Several studies have found that targeted therapy achieves satisfactory effects only in a subset of RCC patients^6,20,21^. Fukuda et al. assessed how tumor thrombus height changed following targeted therapy and found that approximately 20% of patients experienced a steady increase in tumor thrombus height throughout treatment^22^. Notably, they investigated the relationship between the effects of targeted therapy on tumor thrombi and primary renal tumors. While these two variables trended toward correlating, they failed to reach statistical significance^22^. In addition, Niu et al. discovered that immune checkpoint inhibitors were potently effective on primary renal tumors, but not on tumor thrombi, in three patients treated with immunotherapy^17^. These findings may be due to differences in genomic features and tumor microenvironments between VTT and primary RCC. Mutational heterogeneity has been found in RCC between the primary tumor and the tumor thrombus in an individual patient^7,8^. Warsow et al. further suggested that despite mutational heterogeneity, no additional mutations are required for tumor thrombus formation. Rather, this process could be driven by pre-existing subclones of the primary tumor^11^.

For molecular classification and prognosis prediction of ccRCC patients with VTT, Kotlar et al. developed a microRNA-based risk classifier containing miR-21, miR-126, and miR-221^23^. Gu et al. determined the prognostic value of the systemic immune inflammation index, which was defined as platelet count × neutrophil counts/lymphocyte counts^24^. To the best of our knowledge, no published studies on gene expression differences between RCC and matched VTT have been undertaken using integrative transcriptome and proteome analyses comparing normal, tumor, and thrombus tissues from ccRCC patients. In our study, two clusters of dysregulated proteins were identified according to their changing trends in thrombus, tumor, and normal tissues. Combining the results of Wang’s and our own datasets revealed progressive downregulation of RAB25 and GGT5 from normal tissue to tumor to thrombus, suggesting their consistent suppressive roles in the development of both thrombi and tumors. Endosomal recycling of growth factor and adhesion molecule receptors to the plasma membrane prevents them from becoming degraded and recycles their functions, thereby significantly influencing cancer progression^25^. Cheng et al. claimed that the loss of the endosomal recycling protein Rab25 could promote tumor angiogenesis by modulating VEGF-A and VEGFR-1 expressions^26,27^. Low GGT5 (a protein with enzymatic activity) expression in renal tumors and thrombus tissues has also been reported in published research^28^. However, GGT5 regulation and its role in cancer remain unclear.

Interestingly, comprehensive data from Wang’s and our own datasets indicate that SHMT2 might enhance ccRCC tumorigenesis while impairing tumor thrombus invasion, whereas CADM4 possibly exerts opposite effects on tumorigenesis and thrombus invasion. These findings suggest that SHMT2 and CADM4 play opposing context-dependent roles in cancer progression. Their distinctive properties for discriminating thrombus, tumor, and normal tissues make these molecules of great research value for ccRCC subtyping and treatment. Usman et al. discovered that SHMT2 is associated with CD8^+^ T cell infiltration in four different human malignancies, including renal papillary cell carcinoma^29^. Nagata et al. showed that CADM4, an immunoglobulin superfamily cell adhesion molecule, is suppressed in ccRCC with vascular infiltration, suggesting that loss of CADM4 is involved in tumor invasion^30^.

Considering that thrombus transcriptome information is not included in the TCGA database, our survival analysis mainly focused on the correlation between prognosis and the mRNA expression levels of the dysregulated genes discovered by our integrative transcriptome and proteome analyses. As shown in Fig. 6A–C, the prognosis correlation was mostly in accord with the expression change trends of dysregulated genes, verified by IHC assays of our ccRCC cohort. The downregulated genes (DEPTOR, DPEP1, NAT8, and SUSD2) in the tumor and thrombus were associated with a worse survival rate when the gene was less expressed in the tumor, and the same correlation was observed for the upregulated genes (PLOD2 and SLC7A5). The above six genes were only found to change in the thrombus and their matched primary tumors compared with normal samples. No difference was observed between thrombus and tumor. This finding suggests that thrombus is predetermined by genomic instability and metastasis-prone features in primary tumors. Although some studies have indicated that the lack of new driver events in thrombi might be due to their rapid extension and/or limited selective pressure in the intravascular space^31^, the underlying mechanism remains unclear.

Regarding the biological activities of these dysregulated genes in ccRCC, Doan et al. demonstrated that DEPTOR was repressed by hypoxia-inducible factors HIF-1/2, and that restoration of DEPTOR confers resistance to mTOR kinase inhibition in ccRCC^32^. In addition, the initial activation, proliferation, and differentiation of CD4^+^ T cells, which modulate the invasion and proliferation of ccRCC, are regulated by cell-intrinsic DEPTOR-dependent interactions^33,34^. Fangshi et al. indicated that an increase in PLOD2 copy number could lead to an increase in CD4^+^ T cell infiltration and affect the immune microenvironment in ccRCC^35^. Elorza et al. showed that HIF2α could increase mTOR complex 1 activity by upregulating the amino acid carrier SLC7A5^36^. You et al. identified NAT8 as a cancer-associated fibroblast (CAF)-related methylation-driven gene that might contribute to CAF infiltration in ccRCC^37^. Furthermore, the ccRCC subtype associated with high NAT8 expression is most responsive to immune checkpoint inhibitors^38^. However, no study has yet reported roles for DPEP1 and SUSD2 in ccRCC tumorigenesis and invasion. Further mechanistic investigations of these molecules will broaden the application scope of our prognostic classifier, including prediction of patient response to targeted therapy or checkpoint immunotherapy and discovery of novel therapeutic targets.

This study has a few limitations. Because of the difficulty in collecting high-quality matched normal, tumor, and thrombus samples, 15 tissue samples from five patients were selected for RNA sequencing and mass spectrometry in our study, which was sufficient to show differences at the sequencing level. However, limited by the sample sizes of the transcriptome and proteome sequencing, the prevalence of some potentially valuable genes did not differ significantly among normal, tumor, and thrombus tissue samples from ccRCC patients. Secondly, the present study was preliminary and aimed to provide a global view of the molecular features of ccRCC with VTT. Further studies are required to reveal the biological functions of these genes and to determine whether these changes in expression trigger tumor thrombus formation.

## Conclusions

This study employed comprehensive transcriptome and proteome analyses of ccRCC patients with VTT to discover distinctive molecular features and pathways relevant to thrombus formation. A six-gene-based prognostic classifier consisting of DEPTOR, DPEP1, NAT8, PLOD2, SLC7A5, and SUSD2 was developed and showed satisfactory performance in predicting the survival of patients with ccRCC. Our findings not only provide vital information for mechanistic research but also reveal novel biomarkers for risk stratification and therapeutic decision-making in patients with ccRCC.

## Supplementary Information

Supplementary Information 1.

Supplementary Table 1.

## Data Availability

The original contributions presented in the study are publicly available. The data of transcriptome sequencing can be found here: [https://ngdc.cncb.ac.cn/gsa-human/s/Mqrh51O3], and the data of proteome sequencing can be found here: [https://ngdc.cncb.ac.cn/omix/preview/PnCKcJg6]. In addition, publicly available datasets were analyzed in this study. The TCGA-KIRC dataset can be found here: [https://portal.gdc.cancer.gov/projects/TCGA-KIRC], and Wang’s external dataset can be found here: [https://www.ncbi.nlm.nih.gov/bioproject/PRJNA596359 and https://www.ncbi.nlm.nih.gov/bioproject/PRJNA596338].
