# Supplementary material for: Integrative transcriptome and proteome analyses of clear cell renal cell carcinoma develop a prognostic classifier associated with thrombus
Source: Sci Rep. 2023 Jun 16;13:9778. doi: 10.1038/s41598-023-36978-5 (PMC10276054; doi:10.1038/s41598-023-36978-5)
Supplement: Supplementary file 1 — Supplementary Information 1. [file 41598_2023_36978_MOESM1_ESM.docx]

**Supplemental Materials and Methods**

**RNA sequencing and mass spectrometry**

Total RNA of tissue samples were extracted using the mirVana miRNA Isolation Kit (Ambion, Austin, TX, USA) following the manufacturer’s protocol. The libraries were constructed using TruSeq Stranded mRNA LTSample Prep Kit (Illumina, San Diego, CA, USA) according to the manufacturer’s instructions. Then these libraries were sequenced on the Illumina sequencing platform (HiSeqTM 2500 or Illumina HiSeq X Ten). Tissue protein extract was digested into peptides with trypsin in tissue samples. The resulting peptides were desalted and concentrated before mass spectrometry by the STAGE-TIP method using a C18 resin disk. The resulting mass spectrometry raw files were submitted to the MaxQuant software version 1.6.17.0 for protein identification and label-free quantification. Known contaminants as provided by MaxQuant and identified in the samples were excluded from further analysis.

**Protein-protein interaction (PPI) network and module analyses**

The investigation of PPIs may aid the identification of protein functions at the molecular level and improve the understanding of various cellular activities, including growth, development, metabolism, differentiation and apoptosis. The Search Tool for the Retrieval of Interacting Genes (STRING; www.string-db.org/) is a database that provides experimental and predicted interaction information. The proteins associated with DEGs were selected according to the STRING database (version 11.0). When the required confidence (score) was >0.9, there was a protein-protein interaction, which was selected to establish the PPI network.

**Data collection and preprocessing of external datasets**

Gene expression profile and clinical data from the TCGA database were downloaded from the University of California Santa Cruz Xena Hub datasets (https://xenabrowser.net/). Data were obtained for 611 samples (tumor tissue and matched normal tissue) and were log2(x+1) transformed. WGTEx gene expression data were also log2(x+1) transformed to allow comparisons to TCGA data. Transformation of Ensembl identifiers and normalization of expression between the TCGA and Ma datasets were also performed prior to subsequent differential analysis. PRJNA596359 and PRJNA596338 transcriptome-sequencing data for 61 tumor-normal pairs and 30 primary-thrombus pairs from 152 Chinese patients with ccRCC provided by Wang et al. was downloaded from the NCBI Sequence Read Archive database and used as the validation dataset.

**Immunohistochemistry staining of tissue microarray**

The formalin-fixed, paraffin-embedded tissue microarray contains 40 ccRCC tumor specimens and their matched normal renal specimens, obtained after partial or radical nephrectomy. Informed consent was obtained from all patients. Immunostaining for targeted proteins was performed using antibodies against human DEPTOR (#20985-1-AP, Proteintech), DPEP1 (#12222-1-AP, Proteintech), NAT8 (#ab197793, Abcam), PLOD2 (#21214-1-AP, Proteintech), SLC7A5 (#28670-1-AP, Proteintech), SUSD2 (#ab121214, Abcam), following the manufacturer’s instructions of a commercial kit (Lab vision, Fremont, CA, USA). The staining score was blindly evaluated by two observers, using a microscope at a magnification of 200×.

The semi-quantitative immunohistochemistry scores were determined on a 4-point scale. Score 0 (negative): no staining or faint/barely perceptible incomplete staining in ≤10% of tumor cells; score 1 (mild): faint/barely perceptible incomplete staining in >10% of tumor cells; score 2 (moderate): weak/moderate complete staining in >10% of tumor cells; score 3 (strong): circumferential complete intense staining in >10% of tumor cells. The manual scoring was independently performed by an experienced pathologist (Yisha Gao) and a senior researcher (Xinxin Gan). Discordant cases were re-evaluated by both observers to establish a consensus score.

**Supplemental Figures**


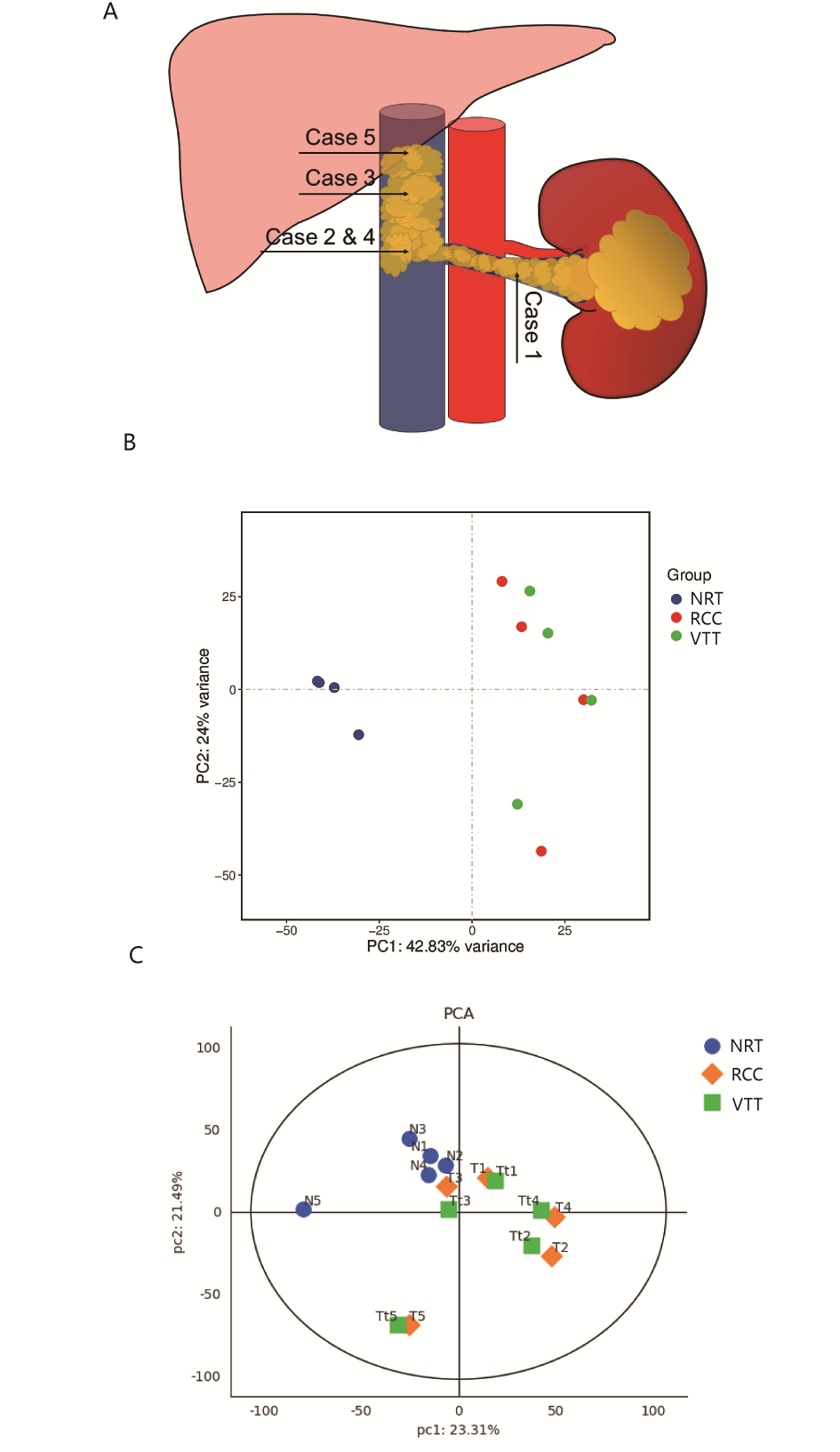


Figure S1. Feature overview of ccRCC associated with VTT. (A) Anatomical diagram of patients with thrombus. (B and C) Three-dimensional partial least squares-discriminant analysis (PLS-DA) score plot of transcriptomic and proteomic data between individual samples.


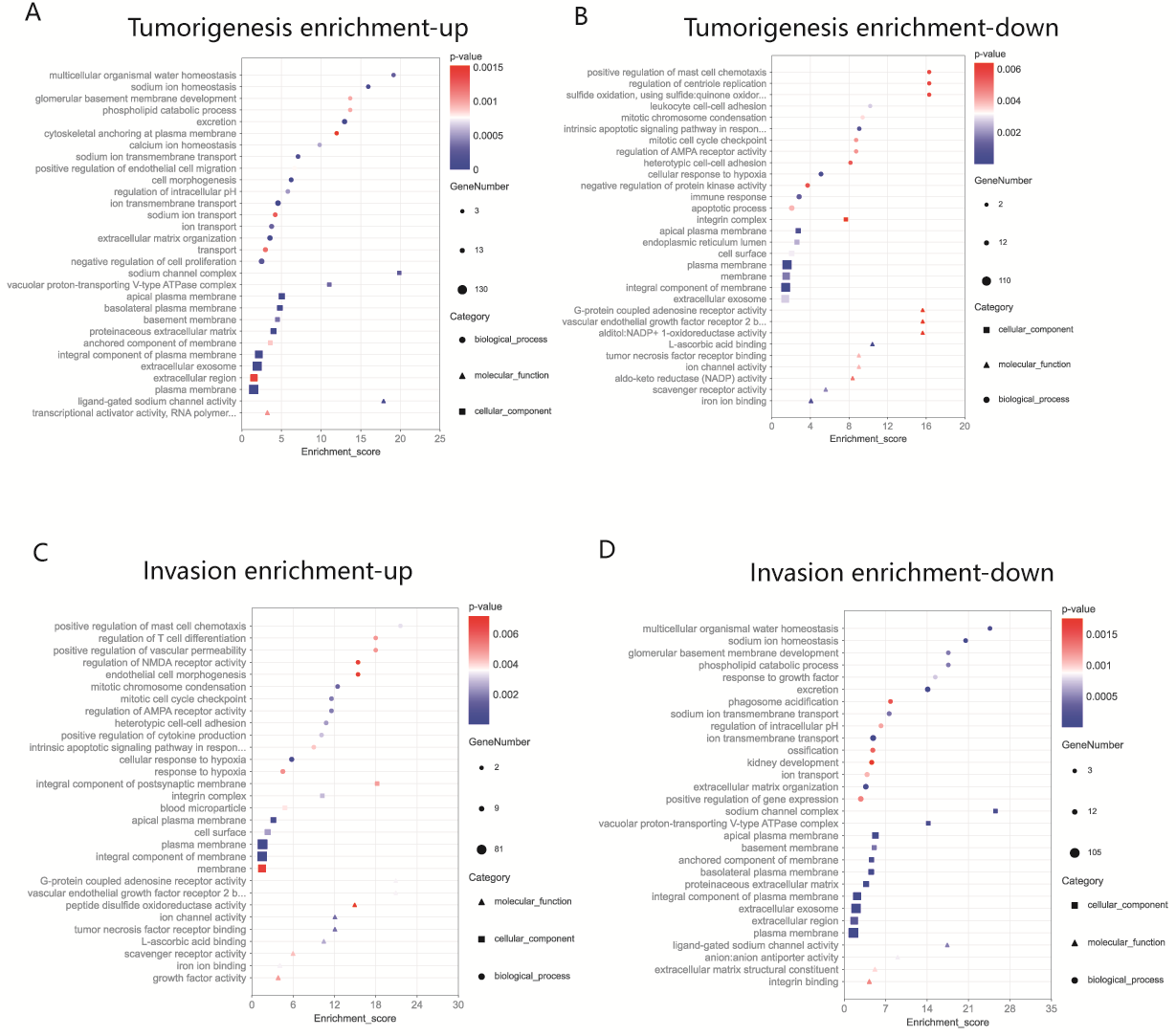


Figure S2. GO analysis of the transcriptomic data. (A and B) The enriched functional annotation of significantly upregulated or downregulated genes in tumorigenesis. (C and D) The enriched functional annotation of significantly upregulated or downregulated genes in thrombus invasion.


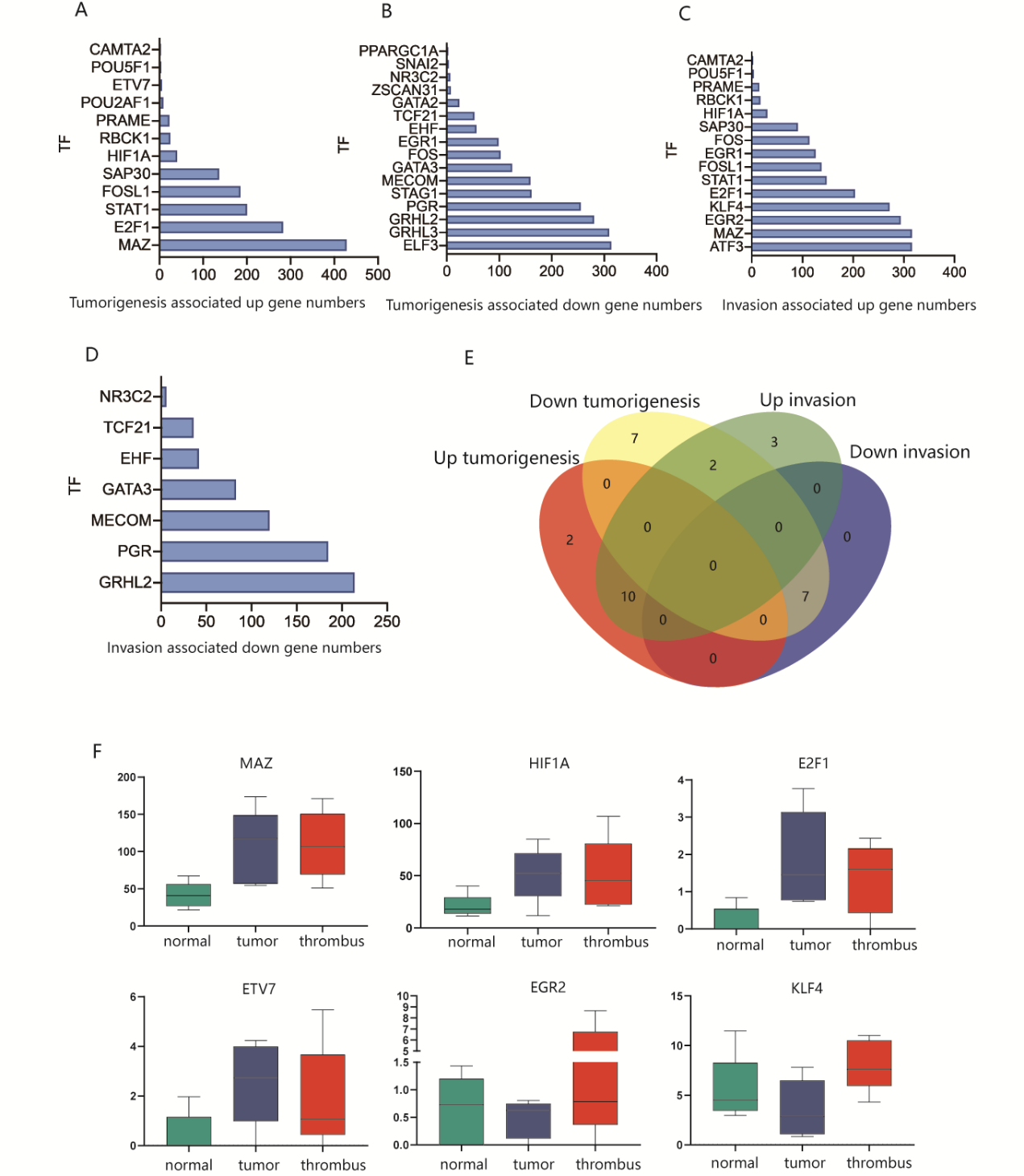


Figure S3. Transcription factor analysis of the transcriptomic data. (A and B) The transcription factors significantly upregulated or downregulated in tumorigenesis. (C and D) The transcription factors significantly upregulated or downregulated in thrombus invasion. (E) Venn diagram to illustrate the consistently and differentially expressed transcription factors in tumorigenesis and thrombus invasion. (F) Expression change trends of the six transcription factors among normal, tumor and thrombus tissue.


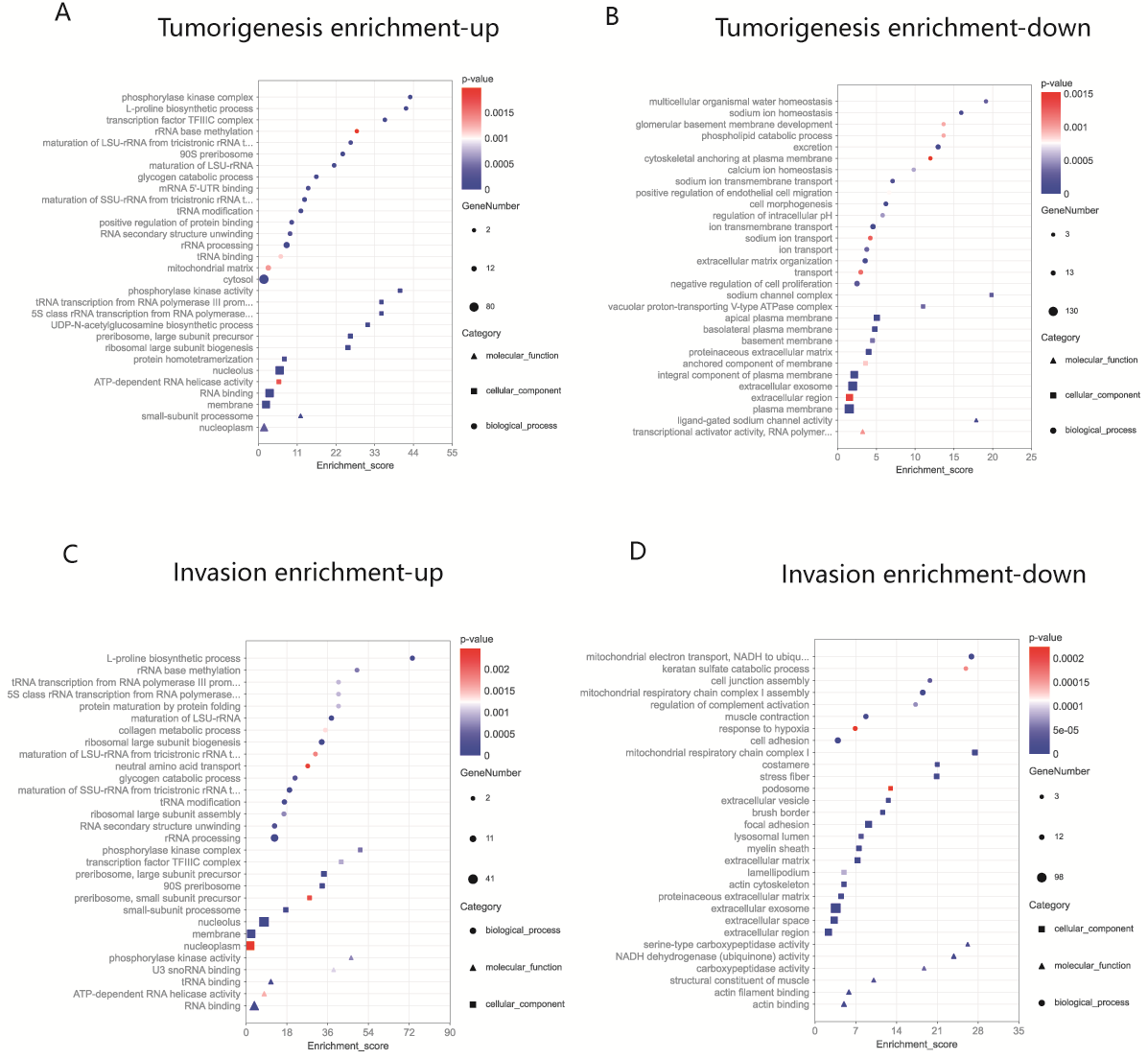


Figure S4. GO analysis of the proteomic data. (A and B) The enriched functional annotation of significantly upregulated or downregulated proteins in tumorigenesis. (C and D) The enriched functional annotation of significantly upregulated or downregulated proteins in thrombus invasion.


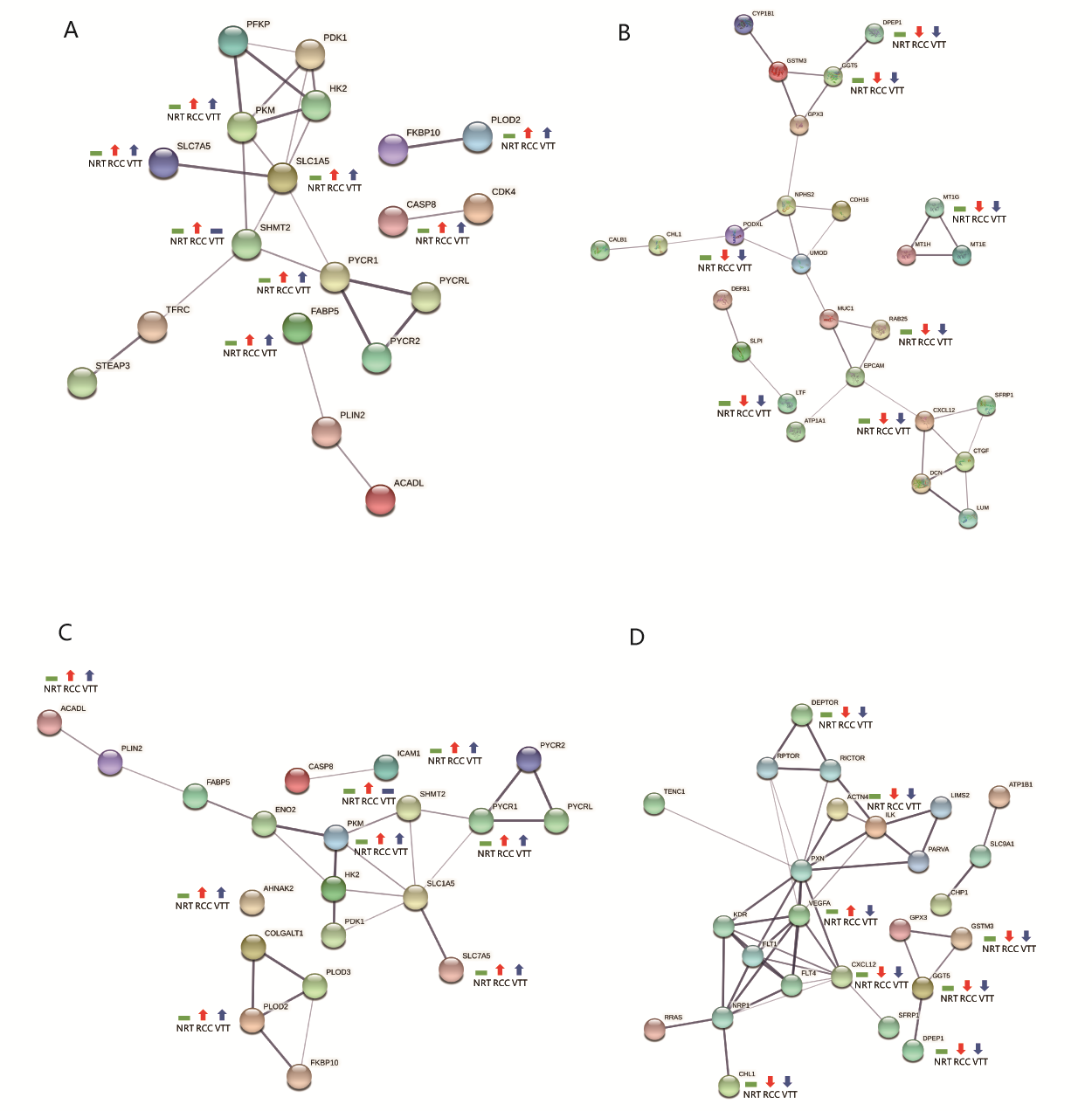


Figure S5. Protein-protein interaction network analysis of the proteomic data. (A and B) The densely connected network constructed with the upregulated or downregulated proteins in tumorigenesis. (C and D) The densely connected network constructed with the upregulated or downregulated proteins in thrombus invasion.


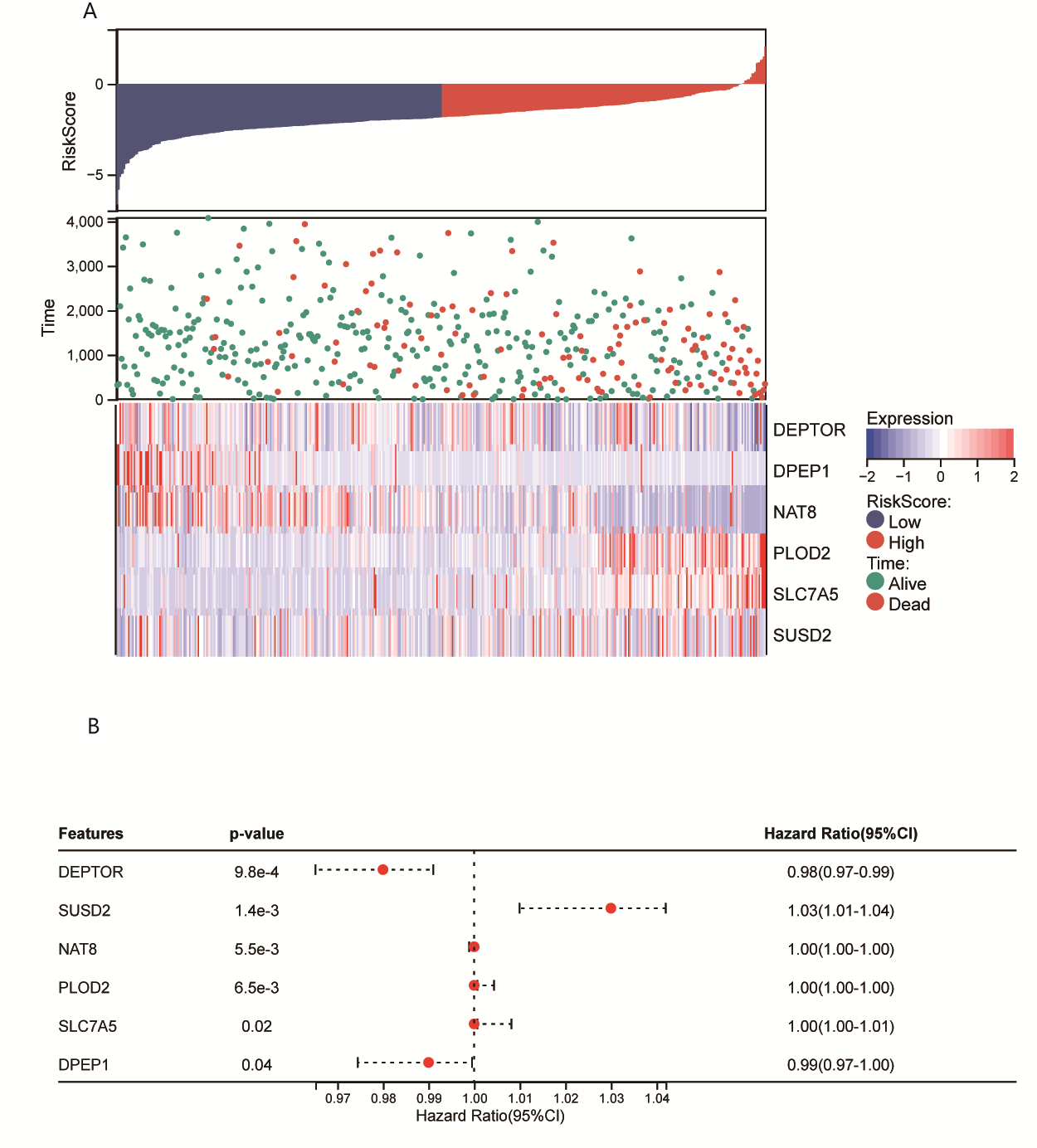


Figure S6. The thrombus-associated gene signature and prognostic classifier. (A) Distributions of risk score and expression profile of the genes in prognostic classifier in patients with different survival time and status. (B) Forest plot of hazard ratios for the genes in prognostic classifier.
