# Supplementary material for: Integrative transcriptome and proteome analyses of clear cell renal cell carcinoma develop a prognostic classifier associated with thrombus
Source: Sci Rep. 2023 Jun 16;13:9778. doi: 10.1038/s41598-023-36978-5 (PMC10276054; doi:10.1038/s41598-023-36978-5)
Supplement: Supplementary file 2 — Supplementary Table 1. [file 41598_2023_36978_MOESM2_ESM.docx]

Table S1. Baseline characteristics of ccRCC patients and their tissue samples.

| No. | Gender | Age (years) | BMI  (kg/m^2^) | Kidney side | WHO/ISUP grade | Renal mass diameter (cm) | Thrombus location | Thrombus length (cm) |
| --- | --- | --- | --- | --- | --- | --- | --- | --- |
| 1 | Male | 53 | 37.2 | Left | II | 4.5 | Renal vein | 2 |
| 2 | Male | 63 | 25.6 | Right | II | 13.5 | Inferior vena cava | 5 |
| 3 | Male | 85 | 28.4 | Right | II-III | 6.2 | Inferior vena cava | 8 |
| 4 | Female | 62 | 21.6 | Right | IV | 9.5 | Inferior vena cava | 5 |
| 5 | Female | 62 | 28.9 | Right | II | 10.5 | Inferior vena cava | 10 |
